# Design and Performance Evaluation of a Deep Ultraviolet LED-Based Ozone Sensor for Semiconductor Industry Applications

**DOI:** 10.3390/mi15040476

**Published:** 2024-03-30

**Authors:** Maosen Xu, Xin Tian, Yuzhe Lin, Yan Xu, Jifang Tao

**Affiliations:** 1 College of Electrical Engineering and Automation, Shandong University of Science and Technology, Qingdao 266590, China; xumaosenld@163.com; 2 College of Electronic and Information Engineering, Shandong University of Science and Technology, Qingdao 266590, China; 3 School of Information Science and Engineering (ISE), Shandong University, Qingdao 266237, China; linyz@sdu.edu.cn; 4 The Key Laboratory of Laser and Infrared System, Ministry of Education, Shandong University, Qingdao 266237, China; tianxin1012@mail.sdu.edu.cn

**Keywords:** gas sensor, ozone detector, UV-LED

## Abstract

Ozone (O_3_) is a critical gas in various industrial applications, particularly in semiconductor manufacturing, where it is used for wafer cleaning and oxidation processes. Accurate and reliable detection of ozone concentration is essential for process control, ensuring product quality, and safeguarding workplace safety. By studying the UV absorption characteristics of O_3_ and combining the specific operational needs of semiconductor process gas analysis, a pressure-insensitive ozone gas sensor has been developed. In its optical structure, a straight-through design without corners was adopted, achieving a coupling efficiency of 52% in the gas chamber. This device can operate reliably in a temperature range from 0 °C to 50 °C, with only ±0.3% full-scale error across the entire temperature range. The sensor consists of a deep ultraviolet light-emitting diode in a narrow spectrum centered at 254 nm, a photodetector, and a gas chamber, with dimensions of 85 mm × 25 mm × 35 mm. The performance of the sensor has been meticulously evaluated through simulation and experimental analysis. The sensor’s gas detection accuracy is 750 ppb, with a rapid response time (*t*_90_) of 7 s, and a limit of detection of 2.26 ppm. It has the potential to be applied in various fields for ozone monitoring, including the semiconductor industry, water treatment facilities, and environmental research.

## 1. Introduction

In traditional semiconductor process control, ozone gas sensors play a crucial role, primarily used for monitoring and regulating the concentration of ozone used in cleaning and oxidation processes [1]. These sensors ensure effective decontamination of chip surfaces during cleaning steps by leveraging the strong oxidizing properties of ozonated water to remove organic pollutants, while avoiding over-oxidation damage. In the oxidation process, sensors control the ozone concentration to optimize the quality of silicon films, especially when growing high-quality films at low temperatures. Additionally, sensor data serve as feedback for automated control systems, adjusting the supply of ozone in real time to maintain process stability and continuity. In such cases, ozone concentrations typically range from several hundred ppm to several thousand ppm. In the concentration range of around 200 ppm, ozone is used for disinfection and deodorization in the hospital and pharmaceutical industries [2]. Ozone gas is very useful for human activity and plays an important role in the natural environment.

At present, there are three main methods for detecting ozone sensors, namely, electrochemical [3], metal oxide semiconductor (MOS) sensors [4], and optical sensors based on absorption in the ultraviolet (UV) region [5]. Electrochemical sensors offer high sensitivity and low cost but can suffer from cross-sensitivity to other gases and require frequent calibration [6]. MOS sensors provide rapid response times and are durable; however, they can be affected by changes in humidity and temperature [7]. Chemiluminescence detectors are highly selective and sensitive but are typically more complex and expensive, making them less suitable for portable or on-site applications [8]. When compared to other types of gas sensors, optical gas sensors offer numerous advantages such as high reliability, rapid response times, excellent selectivity, and cost-effectiveness. The ongoing development of wide-bandgap semiconductor materials, including Ga_2_O_3_ [9], ZnO [10], WO_3_ [11], AlN [12], GaN [13], AlGaN [4], and others, has significantly enhanced the performance of detectors in the UV spectrum, further advancing sensor capabilities.

With the continuous development of UV absorption technology, significant progress has been made in the use of UV optical gas sensors for ozone detection. Maria et al. [14] enhanced the sensitivity of their ozone monitor by increasing the optical path length through back reflection of a light beam, achieving a limit of detection (LOD) of 0.1 ppm. Anderson et al. [15] created a portable ozone monitor utilizing a 15 cm length hollow-core waveguide made of aluminum coated with quartz. In 2012, Aoyagi et al. [16] successfully detected ozone concentrations as low as 0.1 ppm with an accuracy of 0.5% utilizing a gas cell of 20 cm length. Marcus et al. [17] proposed a method for achieving high-sensitivity measurement of ozone concentration by optimizing the optical path length and absorption cross section. It provided crucial technical foundations and guidance for future developments in the field of ozone concentration measurement. Li et al. [18] developed an experimental platform for detecting ozone and NO concentrations in air-insulated switchgear using the principle of ultraviolet differential optical absorption spectroscopy. The NASA ROZE [19] instrument provides high-sensitivity, fasttime-response measurements of ozone via broadband cavity-enhanced UV absorption. In 2022, Barreto et al. [20] unveiled a novel, compact ozone sensor design combining substrate-integrated hollow waveguides with a mini lamp and fiber-optic spectrophotometer, improving design compactness and optical flexibility. However, the device is limited by its narrow detection range and complex structure, potentially hindering its broad usability and ease of deployment. Puga et al. [21] presented a new method for detecting ambient ozone using deep-ultraviolet (DUV) cavity-enhanced absorption spectroscopy (CEAS) with a laser-driven light source (LDLS). The accuracy of sensor is <~2% error and the precision of sensor is 0.3 ppb. However, a notable drawback of this approach is its complexity in structure and operation. Meng et al. [22] designed a miniature, low-power, cost-effective, wide-temperature-range photometric ozone sensor for continuous in situ ambient ozone monitoring. The sensor boasts a detection limit as low as 8 ppb with an impressive accuracy of 0.22%. Preliminary research and analysis reveal that current studies on ozone gas sensors are mainly focused on detecting ozone in the environment (concentrations < 100 ppm) and typically utilize lasers and fiber-optic photometers as basic components, leading to overall sensor complexity and high costs, making them unsuitable for industrial field applications. Moreover, in semiconductor process control where ozone concentrations are higher, electrochemical sensors may encounter issues with absorption saturation, rendering them incapable of functioning correctly. Additionally, other detection methods do not meet the requirements for ozone detection in semiconductor process control applications due to issues with lifespan, reliability, and poor selectivity.

In light of these challenges and advancements, we present an ozone gas sensor based on DUV LED technology. This sensor offers a promising solution for precise ozone concentration monitoring in both ozone generators and semiconductor manufacturing processes. The ozone gas sensor was formed by a DUV LED with narrowband emission spectrum centered at 254 nm, a photodetector, and a gas cell designed for high optical coupling efficiency (the transmittance of light emitted from the light source, passing through the gas chamber, and reaching the detector) of 52%. The ozone gas sensor adopts a discrete structure of light source detector and gas chamber. By optimizing the gas path structure, the influence of pipeline pressure on ozone gas measurement is reduced. We conducted simulations involving optical and pressure modeling on the sensor. We also evaluate the sensor’s performance by assessing factors such as dynamic response, sensitivity, repeatability, and other related metrics. The footprint of the sensor is 80 mm × 25 mm × 35 mm. The gas detection resolution is approximately 750 ppb and the response time (*T*_90_) is 7 s. The LOD in ozone concentrations is 2.26 ppm.

## 2. Design and Simulation of Sensor

**Figure 1:**
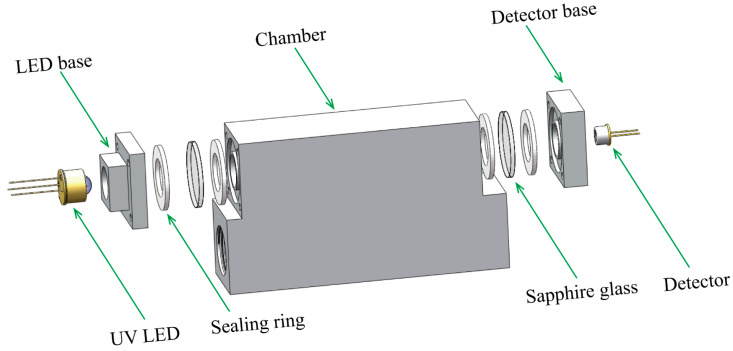
The structure of ozone gas sensor.

The ozone gas sensor is specifically designed for monitoring ozone concentration in ozone generators and chip manufacturing processes. It consists of several key components, including a DUV LED with a central wavelength of 254 nm, a gas chamber, and a photoelectric detector. The primary design objective of this sensor was to achieve high sensitivity for ozone concentration monitoring. The structure of the ozone gas sensor is as shown in Figure 1. The compact design of the sensor facilitates easy installation within industrial processes, with standard industrial gas connections at both ends of the chamber to facilitate integration into industrial field applications. The sensor’s gas path features a design without corners or bends, effectively reducing the surface area exposed to ozone. Due to ozone’s strong corrosive nature, which can lead to contamination of the mirrors of the light source and detector, resulting in inaccurate measurements, it is essential to isolate the light source and detector from the gas. The overall structure is divided into a light source base, a detector base, and a chamber. Sapphire is used as an optical transparent window between the chamber and the base. This is because sapphire has a transmission rate close to 100% in the DUV wavelength range, thereby minimizing loss of light from the light source passing through. To ensure sealing between the base and the chamber, polytetrafluoroethylene (PTFE) sealing rings are used on both sides of the sapphire, combined with the connection of the two structural components to ensure the sealing of the chamber and maintain the integrity of the overall structure. Furthermore, due to its strong antioxidative properties, PTFE serves as the sealing material for the ozone chamber, preventing ozone leakage.

Ozone exhibits an absorption peak at 254 nm, with a smaller absorption peak at 603 nm [23]. Different concentrations of ozone are typically found in mixtures of oxygen and ozone. Therefore, it is important to consider the absorption of oxygen to avoid any interference. The absorption peaks of oxygen are at 150, 688, and 762 nm [24,25]. Due to the well-separated absorption spectra of ozone and oxygen, narrow-band LED light sources can be selected to prevent interference from oxygen during ozone measurements. Therefore, a DUV narrowband light source has been adopted. The light source used in the sensor structure is an LP255 LED from Shenzhen Liupeng Electronics Co., Ltd., Shenzhen, China. It has a center wavelength of 255 ± 5 nm and a bandwidth of 8 nm. The LED has a radiant power of 10 mW. It operates at a supply voltage of 6.5 V and has a viewing angle of 5°. The detector used in the sensor structure is the G280TO1LBP from Hefei Photosensitive Semiconductor Co., Ltd., Hefei, China. It has a peak response wavelength of 255 nm and a responsivity of 0.12 A/W. The spectral response range of the detector is from 240 nm to 280 nm. It has a sensitive area of 1 mm^2^ and is packaged in a TO46 package. The G280TO1LBP detector, selected for its specific peak response wavelength, responsivity, spectral response range, and packaging, is ideal for ozone gas detection in semiconductor manufacturing, crucial for accurate monitoring of ozone concentrations for process control and optimization. The LP255 LED, chosen for its specific wavelength and optical power output, ensuring precise and dependable ozone detection in semiconductor manufacturing environments. Additionally, due to the impact of ultraviolet radiation and humidity on the optical filter, a narrowband light source is utilized to avoid any potential impact on measurements caused by the use of filters.

For a quantitative analysis, it is crucial to take into account the decrease in incident light intensity caused by the absorption of gas molecules, a phenomenon governed by the principles outlined in the Beer–Lambert law [26]:

(1) $$I(\lambda )=I_{0}(\lambda )e^{-\alpha (T,P,\lambda )CL}$$

where *I*_0_(*λ*) refers to the initial light intensity at wavelength *λ*, while *I*(*λ*) refers to the transmitted light intensity after passing through the chamber at wavelength *λ*. *α* is a function that incorporates the effects of temperature (*T*), pressure (*P*), and wavelength (*λ*) on the absorption coefficient. *L* is the optical path length through the gas sample chamber. *C* represents the ozone concentration in parts per million (ppm). This law describes the relationship between the concentration of a substance in a medium and the amount of light absorbed by that substance.

The concentration of ozone can be determined by measuring the incident and transmitted light intensities and applying the Beer–Lambert law formula, which is expressed as follows:

(2) $$C=\frac{1}{\alpha L}\ln\frac{I}{I_{0}}$$

Once the structure of the sensor is fixed, *α* and *L* become constants. Therefore, the concentration of the gas is related only to the ratio of the transmitted light intensity *I* to the initial light intensity *I*_0_. If the coupling efficiency of the chamber has been considered, the transmitted light intensity reaching the detector can be expressed as *I* = *ηI*_0_, where *η* is the optical coupling efficiency of the gas chamber. The sensitivity of the sensor is:

(3) $$S=\frac{dI}{dC}=-\alpha L\eta I_{0}e^{-\alpha CL}$$

Thus, to detect lower-concentration gases, it is essential to enhance sensitivity, with the optical path and optical coupling efficiency optimized to the highest extent possible.

### 2.1. Gas Chamber Design and Assembly of Components

**Figure 2:**
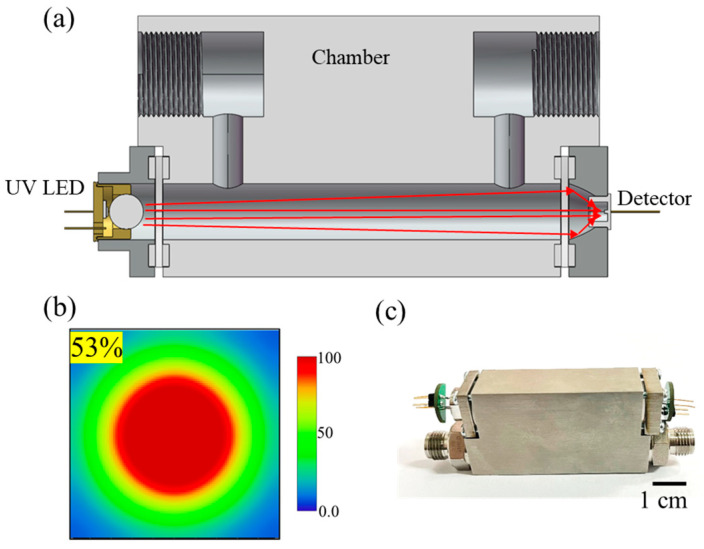
Overview of the gas sensor. (**a**) Cross-section of the gas sensor. (**b**) The light-spot-on detector is simulated by ray trace. (**c**) Photograph of gas sensor.

For high-precision detection of ozone gas in semiconductor process control, the sensor must possess high sensitivity characteristics. The gas chamber is one of the critical components of the sensor. According to the sensitivity calculation Formula (3), for a specific gas, sensitivity is closely related to the optical path length and coupling efficiency, which are determined by the structure of the gas chamber. Therefore, optimization design of the sensor gas chamber structure is required. Given that the LED light source has a small divergence angle of ±5°, in order to minimize light loss during propagation, the sensor chamber adopts a straight-through structure. Simultaneously, it effectively reduces the surface area exposed to ozone. Based on the structural design, an optical simulation model was constructed, and the optical path of the sensor was simulated and optimized using ray tracing methods. As shown in Figure 2a, the detector and light source are positioned directly facing each other. However, due to the larger emitting area of the light source, which measures 20 mm^2^, compared with the smaller receiving area of the detector, measuring only 1 mm^2^, a significant portion of the emitted light fails to reach the detector’s sensitive surface. To address this issue, a compound parabolic concentrator (CPC) structure is employed at the detector location. This structure collects and concentrates the emitted light, thereby increasing the light intensity reaching the detector. Currently, traditional simulation designs typically choose the window position of TO packaging as the detection surface for optical coupling efficiency analysis. However, since the detector chip is actually located at the bottom of the TO package, not all of the light entering the optical window reaches the detector chip. As a result, the actual coupling efficiency is significantly lower than the simulated results. Therefore, by adjusting the aperture and opening angle of the CPC structure, the focal point of the light is shifted downward to align with the effective sensitive area of the detector within the TO package. This adjustment ensures that the light is focused on the detector’s receiving surface. The CPC has a radial aperture of 2.4, an angle of 23°, and a length of 5 mm. As shown in Figure 2b, optical simulations of the gas chamber were conducted using ray-tracing methods. The optical coupling efficiency at the sensitive surface of the detector was 53%. The average optical path length was 80 mm with less than two reflections. The transmission loss per unit distance was 0.58%/mm.

The size of the gas chamber is 85 mm × 25 mm × 35 mm (length × width × height). An overview of the gas chamber is shown in Figure 2c. In the DUV region, aluminum is commonly used as a reflective coating with a reflectivity of 97%. However, ozone’s strong oxidizing nature rapidly causes Al_2_O_3_, leading to the formation of aluminum oxide and a significant decrease in reflectivity. Stainless steel, being resistant to O_3_ corrosion, was chosen as the reflective material for the gas chamber. The gas chamber was polished using a mirror finish to minimize light loss. Stainless steel has a reflectivity of 78% in the deep ultraviolet region, but since the sensor’s reflection occurs only twice, it does not significantly impact the coupling efficiency. Based on the previous fabrication process of the gas chamber, the error between the simulated results and the actual coupling efficiency was less than 1% [27].

### 2.2. Fluid Pressure Simulation

**Figure 3:**
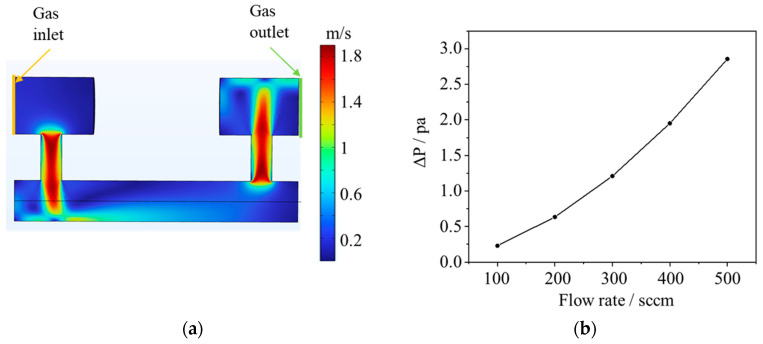
(**a**) Velocity distribution in gas chamber at 500 sccm. (**b**) The curve depicting the relationship between the difference in average pressure inside the optical path and the ambient pressure and the airflow rate.

In practical applications, ozone concentration is influenced not only by concentration ratios but also by other factors such as temperature and pressure. The pressure affects the density of the sample gas, which in turn alters the number of O_3_ molecules absorbed in the detection chamber and impacts the intensity of the light absorbed. Therefore, it is necessary to analyze the overall pressure distribution of the sensor to evaluate its impact on the photodetector. A finite element method (FEM) model was constructed to examine the sensor’s overall pressure distribution. We conducted a parameterized sweep analysis on the flow rate at the sensor inlet, ranging from 100 to 500 sccm with a step size of 100 sccm. The analysis focused on examining the variation in average pressure within the sensor chamber under different flow rates. The gas velocity distribution within the entire gas chamber at a flow rate of 500 sccm at the sensor inlet is illustrated in Figure 3a. Significant fluctuations in velocity occur at locations where there are changes in the aperture of the gas path, while the velocity remains relatively stable at other positions without substantial changes. As shown in Figure 3b, as the flow velocity increases, the difference between the average pressure inside the chamber and the ambient pressure increases. At lower flow velocities, the difference inside the chamber is smaller. However, at higher flow velocities, as the flow rate increases, the pressure difference gradually increases. The maximum difference occurs at a flow rate of 500 sccm, with a maximum pressure fluctuation of 3 Pa, according to the ideal gas law:

(4) PV=nRT

where *P* is the pressure of the gas chamber, *V* is the volumes of the gas chamber, *n* is the number of moles of the gas, *R* is the gas constant, *T* is the absolute temperature of the gas. As the number of moles of gas is directly proportional to the amount of gas, it can be replaced with the gas’s molar concentration *C* multiplied by the volume of the container *V*. Assuming the gas is in a sealed container and the pressure changes from *P*_1_ to *P*_2_, the change in gas concentration can be calculated by the following formula:

(5) $$\frac{C_{2}}{C_{1}}=\frac{P_{2}}{P_{1}}$$

where is the initial gas concentration, and *C*_2_ is the gas concentration when the pressure changes from *P*_1_ to *P*_2_. If pressure is included in the calculation of gas concentration, the equation is derived to correct the change in concentration due to pressure variation, based on the relationship between concentration and pressure as described above:

(6) $$C=\frac{1}{kL}\ln\frac{I}{I_{0}}\times \frac{14.695psi+\Delta P(psi)}{14.695psi}$$

According to the ideal gas law calculation, at a flow rate of 500 sccm, the pressure increases results in a gas concentration increase of approximately 2 × 10^−5^ times. Therefore, for flow rates within 500 sccm, the influence on gas concentration measurements is extremely limited. In practical applications, for ozone gas monitoring at flow rates below 500 sccm, a full-flow detection method can be employed. However, for flow rates exceeding 500 sccm, the flow can be diverted and detected using a bypass low-flow method.

## 3. Experimental Results and Discussions

**Figure 4:**
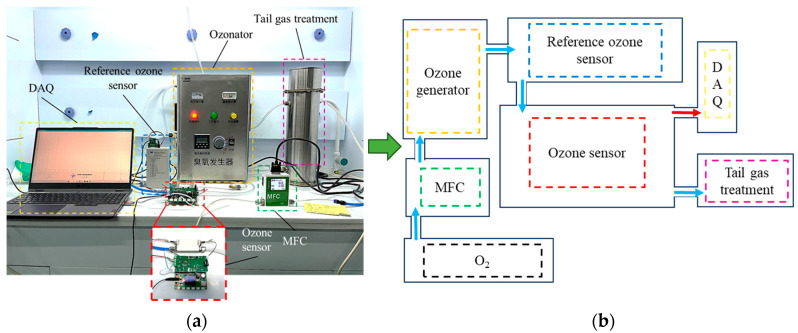
Schematic of the ozone gas sensor test system. (**a**) The overall ozone concentration testing platform consists of a gas source, mass flow controller (MFC), an ozone generator, a standard ozone concentration meter, ozone sensor, ozone tail gas treatment, and a data acquisition system (DAQs). (**b**) The ozone concentration testing process involves the sensor measuring the electrical signal, which is then collected by the DAQs.

To demonstrate the suitability of the sensor for monitoring ozone concentrations in various fields such as the semiconductor industry, water treatment, and ozone research, experimental measurements were conducted at different ozone concentrations ranging from 0% to 0.4%. The experimental setup, as shown in Figure 4, involved feeding O_2_ into the ozone generator to produce ozone at different concentrations by adjusting the electrical power. The generated ozone was calibrated for concentration by a high-precision ozone concentration analyzer before reaching the ozone gas sensor for measurement. The electrical signals measured by the sensor were collected via a data acquisition system (DAQ). The ozone, after measurement, was processed by exhaust tail gas treatment to prevent environmental pollution. The gas flow was fixed at 500 sccm throughout the experiment, and the sensors operated at room temperature of 20 °C. The duty cycle of the pulsed DUV LED was set to 1% at a modulation frequency of 1 Hz. After the sensor reached a stable operational state upon powering, measurements of different gas concentrations were conducted. The ozone concentration was switched every 3 min during the measurement process.

**Figure 5:**
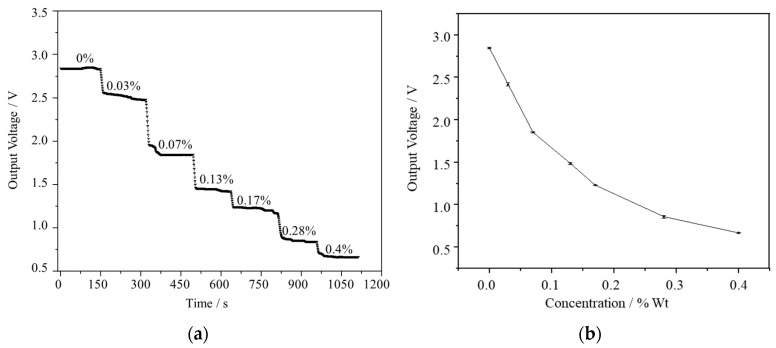
(**a**) The output voltage curve with time. Concentrations of ozone gas, 0–0.4%. (**b**) The sensitivity response curve of the sensor at different concentrations.

**Figure 6:**
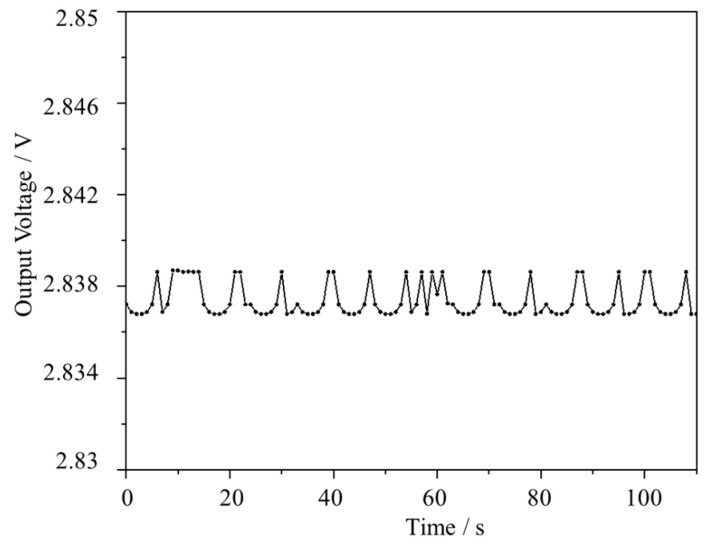
The system noise floor at zero ozone concentration.

The temporal behavior of the sensors was monitored, as shown in Figure 5a, which indicates their dynamic response over time. Through rigorous testing, the sensors exhibited the capability to discern seven distinct concentration points. This ability underscores their sensitivity and precision in detecting variations in environmental conditions. The fluctuations in the sensor signals were quantified using the root mean square (*rms*) method. Figure 6 provides insight into the system noise floor. The statistical analysis of the sensor was conducted over a two-minute duration, encompassing 120 data points, at an ozone concentration level of 0%. During this period, the sensor signals were closely scrutinized to assess their fluctuations. The overall fluctuation in the sensor signals during this time frame was found to be 1.1 mV. This calculation takes into account various factors affecting the signal, including circuit noise, stability of the light source, and potential signal noise caused by gas molecule scattering. This statistical insight provides valuable information regarding the stability and consistency of the sensor’s output, even in the absence of a detectable gas concentration. This metric delineates the minimum discernible signal amidst background noise.

Figure 5b showcases the concentration response curve of the sensor signal, which exhibits adherence to the well-known Beer–Lambert law within the range under investigation. By scrutinizing the slopes of this curve, one can gauge the sensitivity of the sensor. For this analysis, the sensitivity at 0.1% ozone concentration was chosen as the reference point, registering at 1.46 mV per part per million (ppm) for the sensor. Based on the calculated baseline noise, the LOD can be determined by considering the signal-to-noise ratio (SNR). The LOD is typically defined as the lowest analyte concentration that can be reliably detected above the baseline noise level. To determine the LOD, we can use statistical methods to analyze the relationship between the analyte concentration and the corresponding signal response, considering the baseline noise as a reference. By establishing a threshold SNR, typically 3:1, the concentration at which the signal becomes distinguishable from the baseline noise can be identified. Thus, employing three times the *rms* amplitude, denoted as 3σ, as a benchmark for the system’s noise floor, the minimum discernible signal change for the sensor, the LOD values can be extrapolated from experimental data using the equation [28]:

(7) $$\mathrm{LOD}=3\frac{{rms}_{noise}}{slope}$$

where slope refers to the slope value of linear curve fitting of gas sensing response versus gas concentration (ppm), and *rms* noise represents (*rms*) deviation of the baseline [29]. Utilizing the previously computed LOD signal of 3.3 mV, we derived the LOD for ozone concentrations to be 2.26 ppm. Furthermore, drawing from previous testing data, where the signal exhibited a fluctuation of 1.1 mV at the same concentration, we assessed the resolution of the sensor to be approximately 750 ppb. This resolution signifies the sensor’s ability to discern minute changes in ozone concentration levels with a high degree of accuracy and precision, underscoring its efficacy in various environmental monitoring applications.

**Figure 7:**
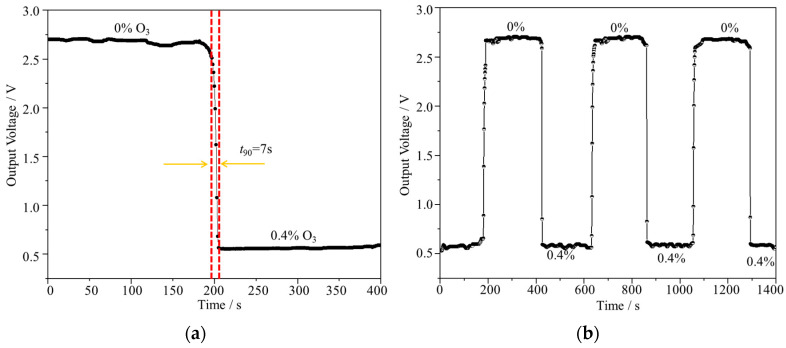
(**a**) The response time of the sensor between pure O_2_ and 0.4% ozone. (**b**) The dynamic response time of the sensor. The concentration of the gas was switched every 4 min between pure O_2_ and 0.4% ozone.

Furthermore, the sensor’s performance was comprehensively assessed through evaluating its response time and repeatability under controlled testing conditions. As shown in Figure 7a, a series of experiments was conducted wherein gas samples were alternated between pure O_2_ and 0.4% ozone. The dynamic response time, represented by *t*_90_ (the duration to achieve 90% of the stable value), was identified as 7 s, emphasizing the sensor’s prompt reaction to environmental changes. In addition, repeatability tests were conducted to ascertain the consistency of the sensor’s performance. As shown in Figure 7b, the full-scale error of merely 0.3% (% FS) during the switching of signals at the same concentration underscores the sensor’s remarkable precision and reliability. Moreover, the signal values obtained for identical concentrations remained consistently stable, further affirming the sensor’s exceptional repeatability and suitability for diverse applications requiring consistent and accurate measurements.

**Figure 8:**
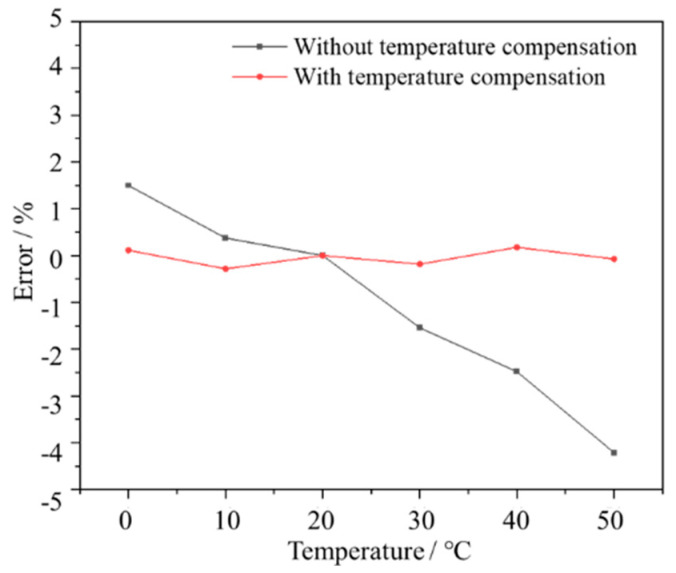
The effect of temperature from 0 to 50 °C on the sensor, for pure O_2_.

As the light source is an LED, its optical power decreases with increasing temperature. Additionally, with rising temperatures, the detector’s noise current increases, thereby resulting in measurement biases in the sensor. Therefore, it is imperative to investigate the influence of temperature on the sensor’s performance. The results are shown in Figure 8. Significant fluctuations in the signal were observed across the tested temperature range spanning from 0 °C to 50 °C. Specifically, the most significant signal fluctuation exceeded 4.2% when measured at 50 °C in comparison to the baseline value recorded at 20 °C. This indicates a considerable temperature drift in the sensor’s response, particularly pronounced at higher temperatures such as 50 °C compared with 20 °C. Consequently, it is imperative to implement temperature-compensation mechanisms to ensure the sensor’s accuracy and reliability across varying temperature conditions. Temperature compensation for the sensor has been accomplished by developing a temperature-output relationship model. Initially, the sensor’s output signals were measured at different concentrations within a 20 °C environment to generate concentration lookup-table data. Subsequently, the sensor underwent testing at various ambient temperatures ranging from 0 to 50 °C, and the output signals at consistent concentrations under different temperature conditions were recorded. These recorded data points constituted the temperature calibration data. These calibration data were utilized to establish a linear model:

(8) $$\Delta V\left(T\right)=29.6-1.351\times T-0.02018\times T^{2}$$

(9) $$V=V_{T}+\Delta V(T)$$

where Δ*V* represents the voltage deviation caused by a certain temperature *T*. The model was employed to compute the deviation in the sensor signal induced by temperature variations. It calculates the calibrated voltage value *V* for a specific concentration based on the actual voltage value *V_T_* at the current temperature, thus alleviating the influence of temperature on the measurements. As shown in Figure 8, after temperature compensation, the fluctuation of the sensor signals significantly decreased, with a maximum fluctuation of 0.28%. The signal fluctuation was reduced by a factor of 15. The measurement accuracy can be kept within 0.3% FS.

## 4. Conclusions

By studying the UV absorption characteristics of O_3_ and combining the specific operational needs of semiconductor process gas analysis, a pressure-insensitive ozone gas sensor has been developed. In its optical structure, a straight-through design without corners has been adopted, achieving a coupling efficiency of 52% in the gas chamber. This device can operate reliably in a temperature range from 0 °C to 50 °C, with an error of only ±0.3% full-scale across the entire temperature range. The sensor’s performance was meticulously evaluated through simulation and experimental analysis. Offering a gas detection resolution of around 750 ppb and a rapid response time (*t*_90_) of 7 s, it achieved LOD in ozone concentrations set at 2.26 ppm, with a measurement accuracy of 0.3% FS. With its robust capabilities, the sensor holds promise for applications in ozone monitoring across diverse sectors such as the semiconductor industry, water treatment facilities, and environmental research.

## Data Availability

Data are contained within the article.
